# Face Experience and the Attentional Bias for Fearful Expressions in 6- and 9-Month-Old Infants

**DOI:** 10.3389/fpsyg.2017.01575

**Published:** 2017-09-20

**Authors:** Kristina Safar, Andrea Kusec, Margaret C. Moulson

**Affiliations:** ^1^Diagnostic Imaging, Hospital for Sick Children Toronto, ON, Canada; ^2^Neurosciences and Mental Health Program, Research Institute, Hospital for Sick Children Toronto, ON, Canada; ^3^MRC Cognition and Brain Sciences Unit, University of Cambridge Cambridge, United Kingdom; ^4^Department of Psychology, Ryerson University Toronto, ON, Canada

**Keywords:** infancy, experience, facial expressions of emotion, emotional face processing, attentional bias, other-race effect

## Abstract

Infants demonstrate an attentional bias toward fearful facial expressions that emerges in the first year of life. The current study investigated whether this attentional bias is influenced by experience with particular face types. Six-month-old (*n* = 33) and 9-month-old (*n* = 31) Caucasian infants' spontaneous preference for fearful facial expressions when expressed by own-race (Caucasian) or other-race (East Asian) faces was examined. Six-month-old infants showed a preference for fearful expressions when expressed by own-race faces, but not when expressed by other-race faces. Nine-month-old infants showed a preference for fearful expressions when expressed by both own-race faces and other-race faces. These results suggest that how infants deploy their attention to different emotional expressions is shaped by experience: Attentional biases might initially be restricted to faces with which infants have the most experience, and later be extended to faces with which they have less experience.

## Introduction

Faces are an important part of the infant's social world. Although newborns display preferences for face-like stimuli (Johnson et al., 1991; Turati et al., 2002) and rudimentary face discrimination ability (Pascalis et al., 1995), their face processing ability undergoes significant development during the first year of life. There is now ample evidence that experience tunes the development of the face-processing system. Experience influences how infants deploy their attention to different faces. For example, soon after birth infants show a looking preference for their mother's face compared to a female stranger's face (Field et al., 1984; Bushnell et al., 1989) and this preference is dose-dependent: newborn infants who see their mother more show a larger preference for her face (Bushnell, 2001). Three-month-old infants show a preference for female over male faces, unless they have a male primary caregiver (Quinn et al., 2002), and a preference for own-race over other-race faces, whereas newborn infants do not (Kelly et al., 2005). These attentional biases are almost certainly driven by greater exposure to female and own-race faces early in the first year of life (Rennels and Davis, 2008; Sugden et al., 2014). These preferences also change over the first year of life: by 9 months, infants show a preference for other-race compared to own-race faces, perhaps because as infants become more efficient at processing own-race faces, they begin to pay more attention to the novel, other-race faces (Liu et al., 2015).

Experience also influences infants' discrimination ability with different face types. Pascalis et al. (2002) investigated infants' ability to discriminate between familiar and novel monkey faces and human faces. Six-month-olds demonstrated discrimination of both monkey and human faces. However, 9-month-olds demonstrated discrimination of human faces but not monkey faces. This latter example of experiential effects is termed perceptual narrowing, which is a process of maintained ability or increasing specialization for faces from familiar categories that comes at the expense of diminished ability for faces from unfamiliar categories (Pascalis et al., 2002; Scott et al., 2007). A similar effect is seen in the processing of own- vs. other-race faces during the first year of life (see Anzures et al., 2013 for a review; Kelly et al., 2007, 2009). For example, Kelly et al. (2007) found that 3-month-old Caucasian infants were able to discriminate between faces of their own race and between faces of other races, whereas 9-month-old Caucasian infants were only able to discriminate between faces of their own race. This specialization for own-race faces that emerges in infancy is a precursor to the “other-race effect” (ORE) in adulthood, where adults demonstrate greater recognition memory for faces of their own race compared to faces of other races (for a review, see Meissner and Brigham, 2001). Interestingly, the emergence of differential processing of own- vs. other-race faces is shaped by experience with face gender. By 3–4 months, infants already show increased discrimination ability for own-race faces when viewing female faces; it was not until 8–9 months that this increased discrimination ability for own-race faces appeared for male faces (Tham et al., 2015).

Relatively fewer studies have examined the effects of experience on processing of emotional faces in the first year of life. This paucity of studies may stem from the theoretical perspective that emotion recognition is universal, implying that experience may not play an important role in shaping this ability (Ekman et al., 1969; Ekman and Friesen, 1971). However, studies that have examined the influence of experience on emotion recognition in infancy show that experience with familiar faces seems to facilitate the ability to discriminate facial expressions (Barrera and Maurer, 1981; Kahana-Kalman and Walker-Andrews, 2001; Walker-Andrews et al., 2011), and alters attentional preferences for particular facial expressions (Bayet et al., 2015). For example, Barrera and Maurer (1981) showed that 3-month-olds were better able to discriminate between happy and sad faces when their mother rather than a female stranger posed the expressions. Similarly, Walker-Andrews and her colleagues showed that 3.5-month-old infants are able to match happy and sad facial and vocal expressions of emotion when posed by their mothers, but not their fathers or unfamiliar females (Kahana-Kalman and Walker-Andrews, 2001; Montague and Walker–Andrews, 2002). Successful matching of unfamiliar faces and voices does not emerge until 5–7 months of age (Walker-Andrews, 1997). Thus, familiarity with particular faces (i.e., mother's face) facilitates early emotion processing ability.

There is also some evidence that differential experience with particular face types (e.g., own- vs. other-race; own- vs. other-species) influences infant emotion processing. Vogel et al. (2012) examined infants' neural responses to congruent and incongruent pairings of happy and sad faces with happy and sad voices using both own-race and other-race faces. They found that 9-month-old infants were sensitive to congruency between the facial and vocal expressions for own-race but not other-race faces, suggesting that emotion processing may be influenced by the increasing specialization for own-race faces that is characteristic of perceptual narrowing. Similarly, (Lewkowicz and Ghazanfar, 2006) found that 4- and 6-month-old infants demonstrate intermodal matching of other-species (i.e., macaque) facial expressions and vocalizations, but 8- and 10-month-old infants do not. More recently, however, Flom et al. (2009) showed that 6-, 18-, and 24-month-old infants all show evidence of intermodal matching of canine facial and vocal expressions (i.e., aggressive vs. nonaggressive expressions). This suggests that ability with other-species emotional expressions does not decline (Flom et al., 2009). Thus, further research is necessary to determine how experience influences processing of emotion expressed by familiar and unfamiliar face types.

The goal of this study was to examine further the influence of experience on infant emotion processing using a different metric than most previous studies—specifically, how infants deploy their attention to different emotional expressions. Young infants show a spontaneous preference for happy facial expressions (Wilcox and Clayton, 1968; LaBarbera et al., 1976; Farroni et al., 2007), but a shift in attentional preference seems to occur by 7 months of age. Specifically, 7-month-old infants consistently show a spontaneous looking preference for fearful over happy facial expressions (Nelson and Dolgin, 1985; Ludemann and Nelson, 1988; de Haan and Nelson, 1998; Kotsoni et al., 2001; Peltola et al., 2009). Consistent with their looking behavior, 7-month-olds also demonstrate greater heart rate deceleration (Peltola et al., 2011), and a larger Nc—an event-related potential (ERP) component that reflects attentional allocation to salient stimuli (Courchesne et al., 1981)—in response to fearful than happy expressions (Nelson and de Haan, 1996; de Haan and Nelson, 1998; de Haan et al., 2004; Leppänen et al., 2007; Moulson et al., 2009; Peltola et al., 2009). More recent evidence suggests that this attentional bias for fearful expressions may emerge earlier than 7 months of age (Forssman et al., 2014; Yrttiaho et al., 2014; Heck et al., 2016, 2017). Although there is debate about what drives the emergence of the attentional bias for fear, it has been increasingly viewed as an index of infants' developing emotion processing ability (Leppänen and Nelson, 2012).

No studies have yet examined whether asymmetrical experience with different face types influences the attentional bias for fearful expressions. However, a recent study from Bayet et al. (2015) found that the preference for happy faces that is present earlier in the first year is modified by face gender. Specifically, 3.5-month-old infants showed a looking preference for smiling over neutral expressions when expressed by female faces, but not when expressed by male faces (Bayet et al., 2015). This finding suggests that the way in which infants deploy their attention to different facial expressions may be modified by familiarity with the types of faces expressing the emotions.

In the current study, we examined whether experience with particular face types (own- and other-race faces) influences 6- and 9-month-old infants' attentional looking preference for fearful facial expressions. Infants viewed own-race and other-race fearful expressions paired with happy expressions posed by the same individual. Their spontaneous looking preferences were measured. We chose to test infants at 6 and 9 months of age based on literature indicating an effect of experience on intermodal matching of emotions (Vogel et al., 2012), while keeping in consideration that increased attention to fearful faces becomes robust sometime between 5 and 7 months of age (Peltola et al., 2008, 2009; Forssman et al., 2014; Yrttiaho et al., 2014; Heck et al., 2016, 2017). Previous literature demonstrating that emotion processing is enhanced for familiar faces (e.g., mother's face: Barrera and Maurer, 1981; Kahana-Kalman and Walker-Andrews, 2001; Montague and Walker–Andrews, 2002) and familiar face types (e.g., female faces; Bayet et al., 2015) would lead us to hypothesize that infants will display an attentional bias for fearful expressions only for own-race faces. In contrast, if infants' deployment of attention to different facial expressions is influenced by perceptual narrowing in the same way that bimodal matching of emotions is (Vogel et al., 2012), we might expect 6-month-olds to show a fear preference for both own- and other-race faces and 9-month-olds to show a fear preference only for own-race faces.

## Methods

### Participants

Thirty-three 6-month-old infants (*M* age = 196.45 days, *SD* = 10.52, 19 boys) and 31 9-month-old infants (*M* age = 289.13 days, *SD* = 12.30, 16 boys) participated. All infants were born within (±) 4 weeks of their due date. Per parent report, none of the infants had been diagnosed with visual impairment or clinical disorders (e.g., pervasive developmental disorders, fetal alcohol spectrum disorders). All participants were Caucasian. An additional 13 infants were tested, but their data were eliminated from analysis due to: (a) infant fussiness (*n* = 10), (b) experimenter error (*n* = 1), (c) side-bias during testing (> 95% looking time to one side across all four trials; *n* = 1), or (d) twin with another infant in the study (*n* = 1).

### Stimuli and procedure

Color photographs of four female faces displaying the facial expressions happiness and fear were drawn from the MacBrain Face Stimulus Set (Tottenham et al., 2009). Only high intensity (i.e., open-mouthed) versions of each emotion were used. To ensure that the particular faces we chose expressed emotions of similar intensity, we examined validity ratings for the happy and fearful expressions of each face. Adults' accuracy at identifying the happy expression averaged 97.5% for the two Caucasian models and 99.5% for the two East Asian models; adults' accuracy at identifying the fearful expression averaged 61% for the two Caucasian models and 72% for the two East Asian models (Tottenham et al., 2009). These accuracies were all significantly above chance. Thus, happy and fearful emotions expressed by both Caucasian and East Asian models were highly recognizable.

All appointment times were scheduled when infants were most active and alert, as reported by the primary caregiver. The Research Ethics Board (REB) of Ryerson University approved the current study. Prior to the start of the study all the parents of participants provided written informed consent. Infants participated in a visual paired-comparison (VPC) task. The infant was seated on the parent's lap facing a computer screen. Parents wore a sleep mask during the task, in order to avoid the possibility of parent behavior influencing infant looking behavior. A video camera situated directly above the computer screen captured infant looking behavior. The video signal was projected onto a second computer screen, which allowed the experimenter to monitor infant looking behavior online during the course of the experiment, such that trials were started only when the infant was looking at the screen. Following the VPC task, parents completed a brief questionnaire assessing the infant's exposure to Caucasian and East Asian faces (see Appendix) and reported the identity and ethnicity of up to six caregivers.

**Figure 1:**
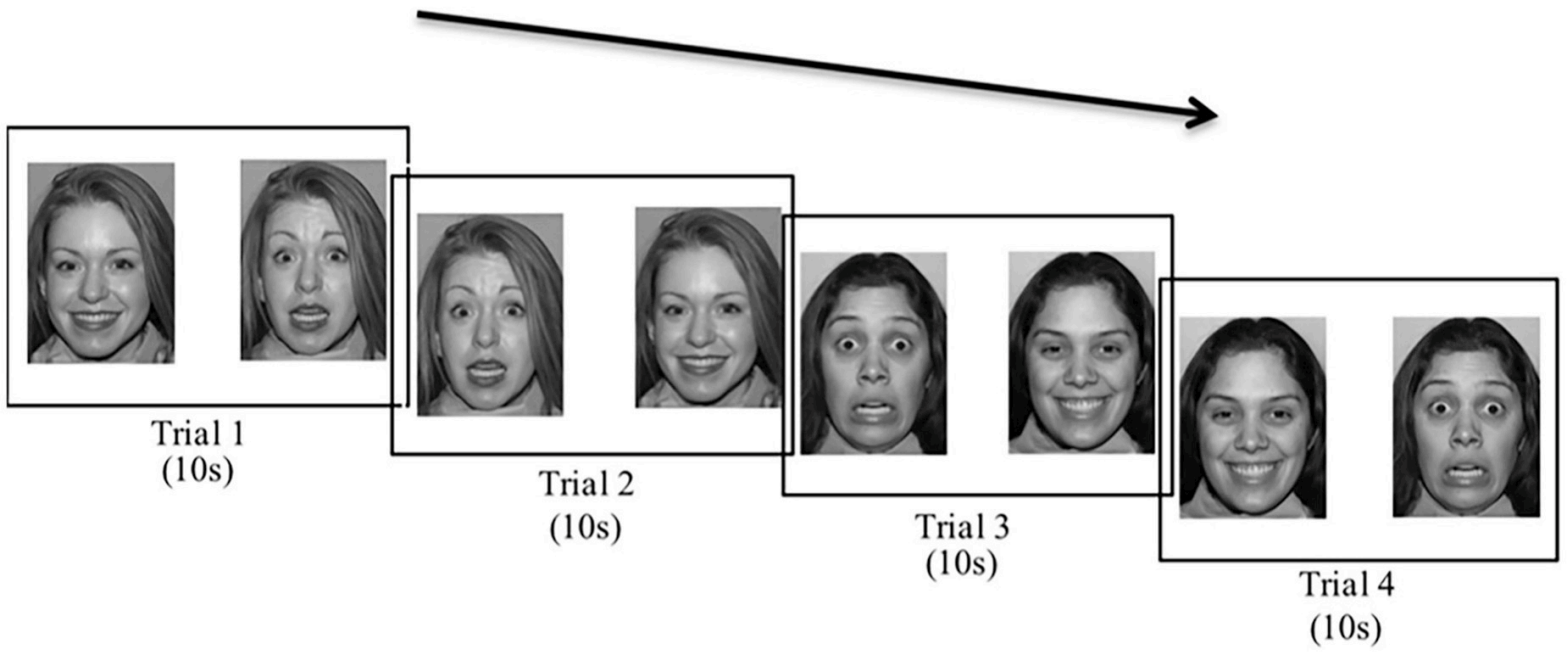
VPC task (own-race Caucasian faces). Infants saw all four 10 s trials. Due to publishing restrictions these are not the exact Caucasian faces that infants were shown.

Two versions of the task were created, one with two Caucasian (own-race) faces and one with two East Asian (other-race) faces. Seventeen 6-month-old infants saw own-race faces and sixteen 6-month-old infants saw other-race faces. Fifteen 9-month-old infants saw own-race faces and sixteen 9-month-old infants saw other-race faces. We used a between-subjects design (i.e., infants saw only own-race faces or only other-race faces) because we were concerned about potential carry-over effects from own-race faces to other-race faces, and vice versa. In particular, it seemed possible that infants might change their deployment of attention over repeated viewings of happy and fearful faces, and this might obscure any initial spontaneous preferences. The VPC task was run using E-Prime (Psychology Software Tools^1^). Infants saw four trials that each lasted for a fixed length of 10 s from the onset of the stimulus. Each trial consisted of one female face expressing happy and fearful facial expressions presented side-by-side on the computer screen. From a viewing distance of 60 cm, each face image subtended ~19 × 24 degrees of visual angle. The first pair of trials showed the same female face, with the left/right position of the happy and fearful expressions reversed from trial 1 to trial 2. The second pair of trials showed a second female face, with the left/right position of the happy and fearful expressions reversed from trial 3 to trial 4 (see Figure 1, faces shown are permitted to be published in scientific journals, Tottenham et al., 2009). Which female face was shown in the first vs. second pair of trials was counterbalanced across infants. Whether the fearful face appeared on the left vs. the right first was also counterbalanced across infants. Between trials, an attention-getting stimulus (a bouncing ball) appeared in the center of the screen to redirect the infant's attention to the screen. The experimenter and a research assistant blind to condition and left-right position of the fearful facial expression coded infant looking time offline. Trials were coded frame-by-frame at 30 frames/second using Adobe Premiere Pro (v5.5). Inter-observer reliability was *r* = 0.88 based on 20% of total infant looking time data.

## Results

Proportion looking time to the fearful facial expression was calculated for each trial [looking time to fearful/(looking time to fearful + looking time to happy)] and averaged across the four trials.

A 2 (age: 6 vs. 9 months) × 2 (condition: own-race vs. other-race) ANOVA was conducted on mean proportion looking time to the fearful facial expression. There were no main effects of age [*F*_(1, 60)_ = 0.323, *p* = 0.572, η_*p*_^2^ = 0.005] or condition [*F*_(1, 60)_ = 0.227, *p* = 0.635, η_*p*_^2^ = 0.004]. However, there was a significant interaction between age and condition [*F*_(1, 60)_ = 4.88, *p* = 0.031, η_*p*_^2^ = 0.075], indicating that 6- and 9-month-old infants performed differently depending on whether they viewed own-race compared to other-race faces. To follow up this interaction, a simple effects analysis (Dunn-Šidak corrected) was conducted to compare the fear preference for own-race vs. other-race faces separately in each age group. Six-month-olds demonstrated a larger fear preference when viewing own-race compared to other-race faces that was marginally significant (*p*_*corr*_ = 0.058). Nine-month-olds, in contrast, did not demonstrate a difference in the size of their fear preference when viewing own-race compared to other-race faces (*p*_*corr*_ = 0.232).

**Figure 2:**
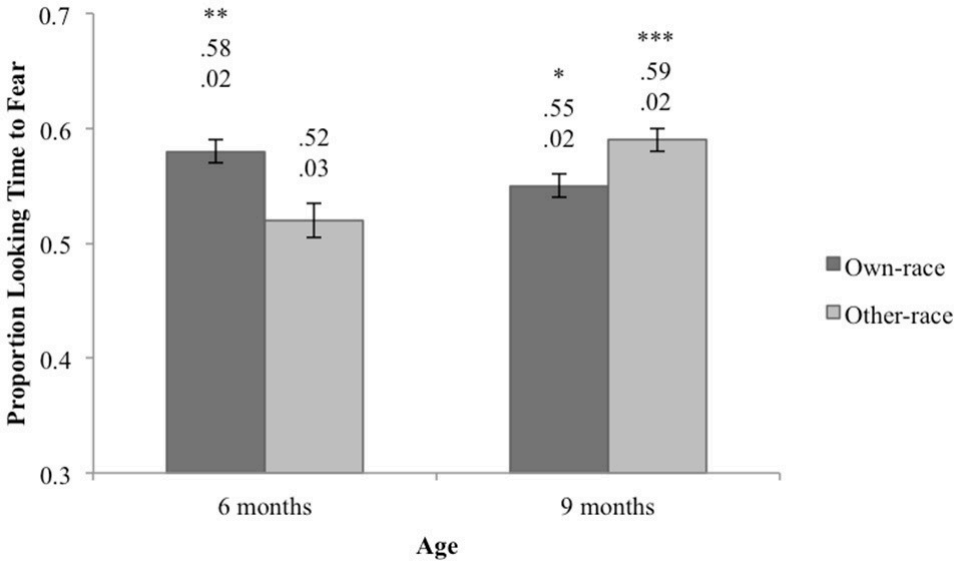
Infant mean proportion looking time to fear. The dark gray bars represent infants who viewed own-race Caucasian faces. The light gray bars represent infants who viewed other-race East Asian faces. Error bars represent standard error of the mean. ^*^p < 0.05; ^**^p < 0.01; ^***^p < 0.001.

To determine which age group(s) in which condition(s) showed a fear preference that was significantly greater than chance, one-sample *t*-tests against chance were then conducted separately by age and condition (Benjamini-Hochberg corrected; see Figure 2). Six-month-old infants who viewed own-race faces demonstrated a significant looking preference for fearful facial expressions compared to happy facial expressions [*M* = 0.58, *SD* = 0.09, *t*_(16)_ = 3.85, *p*_corr_ = 0.002, two-tailed, *d* = 0.93]. Fourteen of these 17 infants showed a preference for the fearful expression that was greater than 50%, *p* = 0.013 using a binomial test. In contrast, 6-month-old infants who viewed other-race faces did not demonstrate a significant looking preference for fearful facial expressions [*M* = 0.52, *SD* = 0.12, *t*_(15)_ = 0.74, *p*_corr_ = 0.471, two-tailed, *d* = 0.18], and only 11 of 16 infants showed a preference for the fearful expression greater than 50%, binomial probability, *p* = 0.21. Nine-month-old infants who viewed own-race faces showed a significant looking preference for fearful facial expressions compared to happy facial expressions [*M* = 0.55, *SD* = 0.07, *t*_(14)_ = 2.39, *p*_corr_ = 0.041, two-tailed, *d* = 0.62], and 11 of the 15 infants showed a fear preference greater than 50%, *p* = 0.118 using a binomial test. Similarly, 9-month-old infants who viewed other-race faces showed a significant looking preference for fearful facial expressions compared to happy facial expressions [*M* = 0.59, *SD* = 0.06, *t*_(15)_ = 5.43, *p*_corr_ = 0.0004, two-tailed, *d* = 1.37], with 14 of 16 infants showing a fear preference greater than 50%, (binomial probability, *p* = 0.004).

### Face exposure

We created two subscales based on the exposure questionnaire. Subscale 1 reflected exposure to Caucasian faces. Subscale 2 reflected exposure to East Asian faces. Questions were reverse-scored as necessary, such that for each scale 1 = little exposure to faces of this type and 4 = much exposure to faces of this type. We conducted a 2 (age: 6 vs. 9 months) × 2 (face type: Caucasian vs. East Asian) ANOVA. We found a significant main effect of face type: Parents reported that their infants received significantly more exposure to Caucasian faces (*M* = 3.76, *SD* = 0.44) than East Asian faces (*M* = 1.42, *SD* = 0.52), *F*_(1, 59)_ = 508.52, *p* < 0.0000001, $\eta_{p}^{2}$ = 0.896. There was no main effect of age, or an age × face type interaction *F*_(1, 59)_ = 0.665, *p* = 0.418, $\eta_{p}^{2}$ = 0.011. Thus, exposure to Caucasian (own-race) and East Asian (other-race) faces did not differ for 6- vs. 9-month-old infants.

We also examined the race of the three most frequently experienced caregivers for each infant. Jayaraman et al. (2015) found that across the first year of life, the three most frequently experienced individuals accounted for more than 80% of all face exposures on average. For all infants in this study, the most frequently experienced caregiver was Caucasian (in all cases, it was the infant's mother). The second most frequently experienced caregiver was Caucasian for all infants except one, whose father self-identified as three-quarters Caucasian and one-quarter Aboriginal. The third most frequently experienced caregiver was Caucasian for all infants except two; one infant's third caregiver was a grandmother who was half Caucasian and half Aboriginal and one infant's third caregiver was a nanny who was East Asian^2^. These findings, combined with the findings from our brief exposure questionnaire, suggest that all of the infants who participated in this study had highly homogenous face experience consisting of primarily own-race faces.

## Discussion

The current study is the first to investigate whether experience with own- and other-race faces influences how infants deploy their attention to different facial expressions at 6 and 9 months of age. Six-month-old infants demonstrated a looking preference for fearful facial expressions compared to happy facial expressions when expressed by own-race faces, but not when expressed by other-race faces. In contrast, 9-month-old infants demonstrated a looking preference for fearful expressions when expressed by both own-race and other-race faces.

The current findings are consistent with previous literature demonstrating that experience with familiar faces (e.g., mother) and face types (e.g., female faces) influences emotion processing (Barrera and Maurer, 1981; Kahana-Kalman and Walker-Andrews, 2001; Montague and Walker–Andrews, 2002; Walker-Andrews et al., 2011; Bayet et al., 2015). In particular, our finding that 6-month-olds show an attentional bias for fear only when expressed by a familiar face type (own-race faces) echoes the finding that 3-month-olds show an attentional bias for happy only when expressed by another familiar face type (female faces; Bayet et al., 2015). Although the particular preference being examined differs due to the different ages in the two studies, these parallel findings suggest that attentional deployment to facial expressions is modified by the face experiences that infants accumulate during their daily lives.

Previous studies have reported significantly more exposure to own-race than other-race faces. Sugden et al. (2014) found that 1- and 3-month-old infants' face exposure was highly biased toward own-race faces: 96% of time spent exposed to faces consisted of own-race face exposure. Similarly, Rennels and Davis (2008) reported that ~92% of the time infants are exposed to same-race caregivers. Exposure data from the current study indicate that infants in this sample had similarly homogenous own-race face experience. Why might this heavily biased exposure to own-race faces affect the attentional bias for fearful facial expressions at 6 months? It is possible that 6-month-old infants more readily attend to fearful facial expressions when expressed by own-race faces because these faces are comprised of perceptual features with which they are familiar (e.g., shape of eyes); thus, infants are better able to detect changes in these facial features, which in turn, facilitates their sensitivity to facial expressions (Kelly et al., 2007). Alternatively, or in addition, it is possible that infants may be greater attuned to facial expressions expressed by frequently experienced faces because they may be relevant indicators of subsequent actions, thus allowing infants to anticipate succeeding interactions and behavior (Kahana-Kalman and Walker-Andrews, 2001). Therefore, it might be advantageous for infants to be sensitive to facial expressions expressed by familiar face types.

A recent account posits that the change in sensitivity to fearful facial expressions in the first year of life reflects functional maturation of neural mechanisms underlying emotion processing and attention (i.e., maturation of connections between amygdala and prefrontal cortex), combined with enhanced experience with fearful facial expressions (Leppänen and Nelson, 2009, 2012). Infants become locomotive around 6–7 months of age; this increase in motor ability is associated with a wider repertoire of facial expressions expressed by caregivers and increased caregiver monitoring on the part of the infant (Campos et al., 1992; Vaish et al., 2008). Leppänen and Nelson (2009, 2012) suggested that the maturation of neural mechanisms that underlie emotion processing might give rise to a sensitive period in development, in which the developing system becomes more responsive to a wider array of facial expressions. Consequently, during this time associations between threatening social objects or situations and fearful facial expressions expressed by their caregivers may be more readily processed (Peltola et al., 2013). The current findings suggest that experience with a particular face type may be necessary for the emergence of the attentional bias to fear. Potentially, this bias is initially tied to familiar face types, but by 9 months it is generalized to faces with which infants have less experience.

It is important to note that our pattern of findings diverges from previous findings of perceptual narrowing in identity discrimination. Specifically, we found that the attentional bias for fear is initially limited to a familiar face category and later extends to an unfamiliar face category. In contrast, identity discrimination is initially observed for both familiar and unfamiliar face categories but later becomes specialized for familiar face categories (Scott et al., 2007). Our findings also differ from Vogel et al. (2012), who used a bimodal matching task to assess the effects of experience on emotion processing, and found results more in line with the typical pattern of perceptual narrowing. It seems likely that different metrics of emotion processing specifically and face processing more generally (i.e., attentional bias vs. bimodal matching vs. discrimination) might be affected by experience in different ways. These disparate findings provide an interesting starting point for future research examining how different metrics of emotion processing might be differentially sensitive to the effects of experience, and how experience might affect identity and emotion processing differently.

It is unclear how specific our pattern of results might be to specific emotions and specific face types examined in the current study. We chose to examine the attentional bias for fear because this finding is well-established in the literature (Nelson and Dolgin, 1985; Ludemann and Nelson, 1988; Peltola et al., 2009). However, by using only one emotion comparison (happy vs. fear), it is impossible for us to determine whether our findings reflect a bias for fear *per se*, or negative expressions in general. This limitation extends to much of the previous literature examining the fear bias in infancy. Future research should investigate how specific the bias is for fearful expressions, which could elucidate the structure of infants' underlying emotion categories (Quinn et al., 2011). Finally, it will be important for future research to replicate the current pattern of results using a comparable sample of East Asian infants presented with the same stimuli. Our conclusions will be considerably strengthened if the same pattern of results is observed.

The current study is the first to examine the role of experience with own- and other-race faces on the attentional bias for fear in infancy. Our findings suggest that the attentional preference for fear might initially be restricted to faces with which infants have the most experience, and later be extended to faces with which they have less experience. This initial attentional preference for fear when expressed by own-race faces is likely due to greater experience with own-race caregivers early in life. Findings contribute to the evolving literature on how face experience influences infant emotion processing.

## Ethics statement

This study was carried out in accordance with the recommendations of the Research Ethics Board (REB) of Ryerson University. All parents of infant subjects gave written informed consent in accordance with the Declaration of Helsinki. The protocol was approved by the REB of Ryerson University.

## Author contributions

KS contributed substantially to conceptualization and study design, data acquisition, data analysis, interpretation of the data, drafting of the manuscript, and final approval of the manuscript to be published. AK contributed substantially to data acquisition, interpretation of the data, critically revising the manuscript for important intellectual content, and final approval of the version to be published. MM contributed substantially to the conceptualization and design of the work, interpretation of data, revising the manuscript critically for important intellectual content, and final approval of the version to be published. All authors (KS, AK, and MM) agree to be accountable for all aspects of the work in ensuring that questions related to the accuracy or integrity of any part of the work are appropriately investigated and resolved.

### Conflict of interest statement

The authors declare that the research was conducted in the absence of any commercial or financial relationships that could be construed as a potential conflict of interest.

## Footnotes

^1^Psychology Software Tools, Inc. [E-Prime 2.0]. (2012). Retrieved from https://www.pstnet.com.

^2^Removing these infants from the analysis did not change the pattern of results.

## Appendix

### Caucasian and east asian face exposure questionnaire

Please indicate the amount of exposure to Caucasian and East Asian faces your infant encounters according to the following scale: *1-Strongly Disagree, 2-Disagree, 3-Agree, 4-Strongly Agree*.

1. My infant sees many Caucasian caregivers on a daily basis…. 1 2 3 4
2. My infant sees many East Asian caregivers on a daily basis…. 1 2 3 4
3. My infant sees many Caucasian caregivers on a weekly basis…. 1 2 3 4
4. My infant sees many East Asian caregivers on a weekly basis…. 1 2 3 4
5. My infant spends the majority of time with Caucasian caregivers…. 1 2 3 4
6. My infant spends minimal time with Caucasian caregivers…. 1 2 3 4
7. My infant spends the majority of time with East Asian caregivers…. 1 2 3 4
8. My infant spends minimal time with East Asian caregivers…. 1 2 3 4
9. My infant interacts often with East Asian faces…. 1 2 3 4
10. My infant interacts predominately with Caucasian faces…. 1 2 3 4
11. My infant interacts minimally with East Asian faces…. 1 2 3 4
12. My infant interacts minimally with Caucasian faces…. 1 2 3 4
